# Genetic origin of patients having spastic paraplegia with or without other neurologic manifestations

**DOI:** 10.1186/s12883-022-02708-z

**Published:** 2022-05-16

**Authors:** Jiannan Chen, Zhe Zhao, Hongrui Shen, Qi Bing, Nan Li, Xuan Guo, Jing Hu

**Affiliations:** Department of Neuromuscular Disease, The Third Affiliated Hospital of Hebei Medical University, 139 Ziqiang Road, Shijiazhuang, Hebei 050000 PR China

**Keywords:** Hereditary spastic paraplegia, Leukodystrophy, Homocysteine remethylation disorders, Hereditary ataxia, Charcot-Marie-tooth atrophy, Genetic analysis

## Abstract

**Background:**

Hereditary spastic paraplegia (HSP) is a group of neurodegenerative diseases characterized by lower-limb spastic paraplegia with highly genetic and clinical heterogeneity. However, the clinical sign of spastic paraplegia can also be seen in a variety of hereditary neurologic diseases with bilateral corticospinal tract impairment. The purpose of this study is to identify the disease spectrum of spastic paraplegia, and to broaden the coverage of genetic testing and recognize clinical, laboratorial, electrophysiological and radiological characteristics to increase the positive rate of diagnosis.

**Methods:**

Twenty-seven cases were screened out to have definite or suspected pathogenic variants from clinically suspected HSP pedigrees through HSP-associated sequencing and/or expanded genetic testing. One case was performed for enzyme detection of leukodystrophy without next-generation sequencing. In addition, detailed clinical, laboratorial, electrophysiological and radiological characteristics of the 28 patients were presented.

**Results:**

A total of five types of hereditary neurological disorders were identified in 28 patients, including HSP (15/28), leukodystrophy (5/28), hereditary ataxia (2/28), methylmalonic acidemia/methylenetetrahydrofolate reductase deficiency (5/28), and Charcot-Marie-tooth atrophy (1/28). Patients in the HSP group had chronic courses, most of whom were lower limbs spasticity, mainly with axonal neuropathy, and thinning corpus callosum, white matter lesions and cerebellar atrophy in brain MRI. In the non-HSP groups, upper and lower limbs both involvement was more common. Patients with homocysteine remethylation disorders or Krabbe’s disease or autosomal recessive spastic ataxia of Charlevoix-Saguenay had diagnostic results in laboratory or imaging examination. A total of 12 new variants were obtained.

**Conclusions:**

HSP had widespread clinical and genetic heterogeneity, and leukodystrophy, hereditary ataxia, Charcot-Marie-Tooth atrophy and homocysteine remethylation disorders accounted for a significant proportion of the proposed HSP. These diseases had different characteristics in clinical, laboratorial, electrophysiological, and radiological aspects, which could help differential diagnosis. Genetic analysis could ultimately provide a clear diagnosis, and broadening the scope of genetic testing could improve the positive rate of diagnosis.

**Supplementary Information:**

The online version contains supplementary material available at 10.1186/s12883-022-02708-z.

## Background

Hereditary spastic paraplegia (HSP) is a syndromic designation for a clinically and genetically heterogeneous group of inherited neurodegenerative disorders in which the main neurological signs are lower-limb spasticity and weakness [1]. To date, more than 80 genes or loci have been published [2, 3]. HSP can be transmitted in autosomal dominant (AD), autosomal recessive (AR), X-linked or mitochondrial maternal modes [4], with 13–40% of cases being sporadic [5]. ADHSP is the most prevalent form of HSP and accounts for approximately 70% of cases. SPG4 (50%), SPG3A (10%), SPG31 (4.5%) and SPG10 (2.5%) are the most common causes of ADHSP [6]. ARHSP is the next largest group of HSP. Mutations in the *CYP7B1* (SPG5), *SPG7* (SPG7), *SPG11* (SPG11), and *ZFYVE26* (SPG15) genes are described as the most frequent causes for ARHSP [6]. Clinically, HSP syndromes are classified as “pure” and “complicated” phenotypes. The pure form is characterized by spastic paraplegia and subtle vibration hypoesthesia in lower extremities, and complicated HSP are those in which pure ones are accompanied by other neurological or non-neurological signs. Neurological features include cerebellar dysfunction, peripheral neuropathy, cognitive impairment, epilepsy, extrapyramidal features, psychiatric disturbances, and brain and spine MRI abnormalities. Non-neurological characteristics are broad and heterogeneous, including ophthalmological abnormalities and skeletal malformations [7–10]. The diagnosis of HSP is based on the presence of bilateral lower limb spasticity (hypertonia, hyperreflexia, and extensor plantar responses) [7, 11]. Although many causal genes have been discovered with the development of genetic testing technology, there are still approximately in 51–71% of all suspected cases of HSP without a genetic diagnosis [2, 5].

Generally, the sign of spastic paraplegia can be seen in a variety of hereditary diseases with bilateral corticospinal tract impairment, leading to HSP overlapping clinically with many other neurodegenerative diseases, such as amyotrophic lateral sclerosis (ALS), leukodystrophy (LD), Charcot-Marie-Tooth atrophy (CMT), hereditary ataxia (HA), familial Alzheimer’s disease (AD), and another rare metabolic disease. Some of these diseases can manifest as not only complicated HSP, such as HA, but pure forms, such as Krabbe’s disease and X-linked adrenoleukodystrophy (X-ALD) [4, 8]. The extensively clinical overlap makes diagnosis more difficult. The study disclosed multiple pathogenic causes of 28 patients with suspected HSP though next-generation sequencing (NGS), and retrospectively analyzed clinic, laboratory, electromyographic and radiological data to summarize identification points.

## Patients and methods

### Patients

A total of 60 indexes with clinically suspected HSP were recruited from the Department of Neuromuscular disease, the Third Affiliated Hospital of Hebei Medical University. Clinically suspected HSP needed to meet the criteria of lower-limb spastic paraplegia (hypertonia, hyperreflexia, and extensor plantar responses) without acquired causes. The physical and auxiliary examinations were confirmed by at least two neurologists. Thereinto, 28 cases were identified through genetic testing or blood test, and detailed clinic, laboratory, electrophysiologic, and radiological characteristics were presented.

### Blood test

All patients underwent routine blood tests to exclude acquired causes of spastic paraparesis, as well as analysis of serum homocysteine (Hcy). One case was performed enzymatic testing for LD.

### Electrophysiology studies

Part of the 28 patients were submitted to nerve conduction studies (NCS, 16/28), electromyography (EMG, 16/28), and sensory evoked potentials (SEP, 18/28) in lower limbs or four limbs to evaluate the presence of axonal or demyelinating neuropathy or motor neuron damage.

### Magnetic resonance imaging

Brain MRI was performed in 20 patients, and thoracic and/or cervical spinal MRI was in 23 patients, using a 1.5/3.0 T system (GE Healthcare, USA).

### Genetic analysis

Genomic DNA was extracted from peripheral blood using the QIAAmp DNA Blood Mini Kit (QIAGEN, Germany). NGS (MyGenostics Inc., Beijing, China), HSP-targeted panel and movement disorder (MD) panel (containing genes of multiple diseases manifested as HSP), were performed, and the list of genes contained in the HSP and MD panels was upload as an additional file. The sequence data were mapped using the BWA and SAMTOOLS software onto the hg19 human genome as a reference. The variants were identified using the wANNOVAR tool, and the probable pathogenic variants were predicted protein function using REVEL tool. The American College of Medical Genetics and Genomics (ACMG) standards were used to evaluate the pathogenicity. Subsequently, the target variants were confirmed using Sanger sequencing in some of the patients and their parents.

## Results

**Fig. 1:**
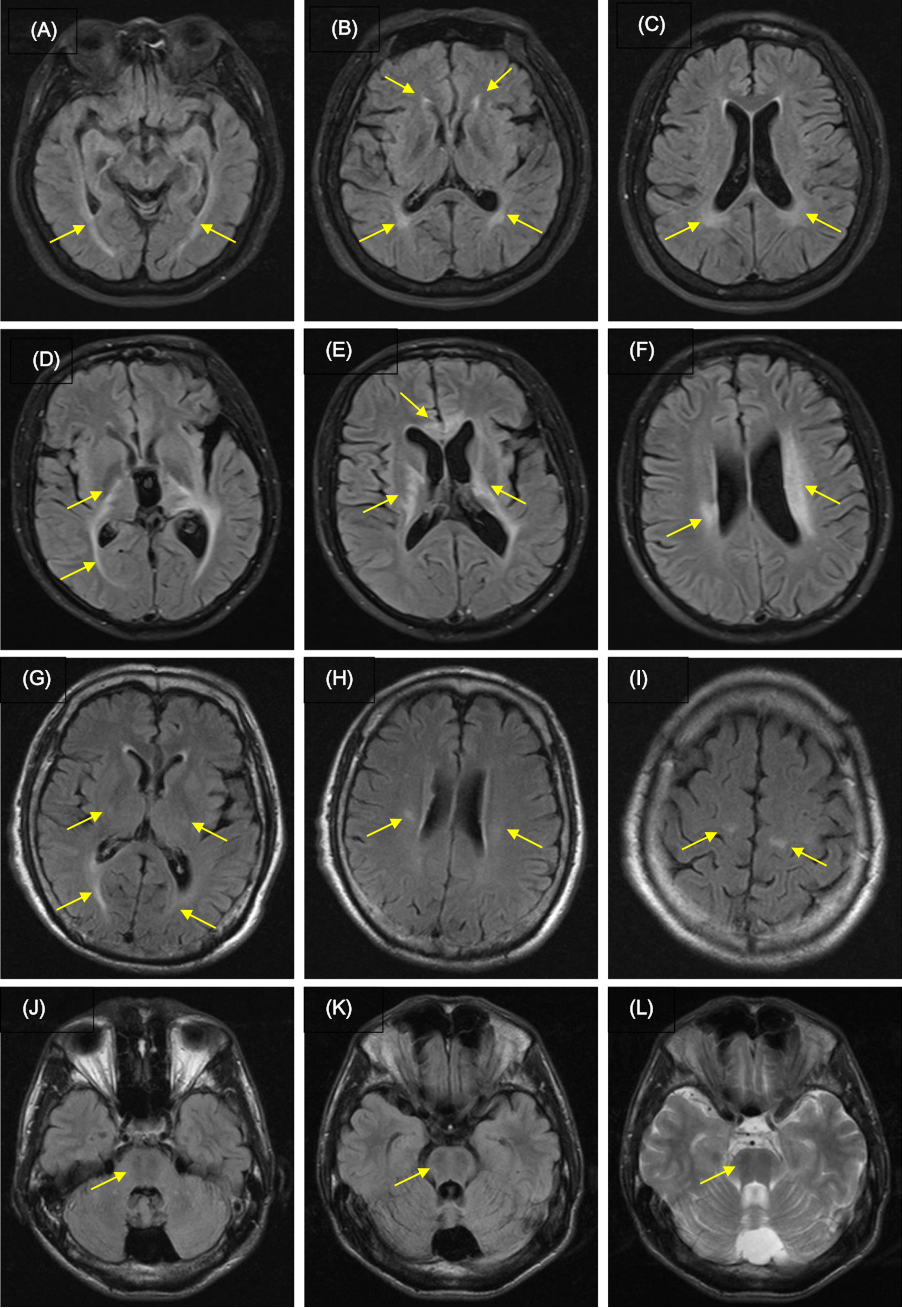
The brain MRIs of four cases. **a** case 14/SPG35, FLAIR sequences showed hyperintensities in the white matter (**A**-**C**) **b** case 22/X-ALD, FLAIR sequences demonstrated bilateral hyperintensities in the deep white matter, corpus callosum and internal capsules (**D**-**F**) **c**. case 25/Krabbe’s Disease, FLAIR sequences showed hyperintensities  in the corticospinal tracts starting in the precentral gyrus and extending to corona radiata, posterior limb of internal capsules, as well as in the terminal areas of white matter (**G**-**I**) **d**. case 27/ARSACS, T2-FLAIR-sequences showed linear hypointensity in the pons and cerebellar atrophy (**J-L**)

Thirty-eight cases were applied in HSP-targeted sequencing, and we subsequently screened out 8 HSP cases with positive rate of 21%. The 30 pedigrees remaining negative and another 21 cases were performed for MD panel, and identified 9 (30%) and 10 (48%) positive cases, respectively (containing HSP and non-HSP cases). Without genetic testing, one case was performed for enzyme detection of LD and had a positive result. A total of 28 positive cases were identified, including 15 cases of HSP, five cases of Hcy remethylation disorders (methylmalonic acidemia/MMA and methylenetetrahydrofolate reductase/MTHFR deficiency), five cases of LD (cerebrotendinous xanthomatosis/CTX, X-ALD, Krabbe’s disease and hypomyelination leukodystrophy-7/HLD7), two cases of HA (autosomal recessive cerebellar ataxia type 8/SCAR8 and autosomal recessive spastic ataxia of Charlevoix-Saguenay/ARSACS), and one case of CMT. We retrospectively analyzed the clinical, laboratory, electrophysiological and radiological findings of 28 cases to help the differential diagnosis (Table 1). Detailed clinical features were shown in Table 2. Brain MRIs of several patients were listed in Fig. 1.

**Table 1:** Clinical, laboratorial, electrophysiological and radiological findings of 28 cases

Clinical types	HSP	MMA/MTHFR deficiency	LD	HA	CMT
cases	15	5	5	2	1
*Clinical findings*					
Age (years)	29.4 (13–62)	22.2 (13–40)	37 (24–68)	30/23	14
AAO (years)	15.5 (3–37)	19.6 (13–34)	35 (24–61)	13/3	12
Upper limbs	3/15	2/5	4/5	2/2	0/1
Lower limbs	15/15	5/5	5/5	2/2	1/1
Cerebral signs	3/15	1/5	0/5	2/2	0/1
Dementia	1/15	1/5	1/5	1/2	0/1
Dysarthria	3/15	1/5	1/5	2/2	0/1
Peripheral neuropathy	3/15	3/5	0/5	1/2	0/1
*Laboratory findings*					
Increased serum Hcy	2/15, ≤25.5 umol/L	5/5, ≥53.1 umol/L	0/5	0/2	0/1
Declined β-galactocerebr-osidase enzyme	–	–	1/1	–	–
*Electro-neurophysiology findings*					
SEP (prolonged central conduction)	8/9	3/3	2/4	1/1	0/1
EMG (extensive spontan-eous potentials)	2/8	0/3	0/2	1/2	1/1
NCS (declined amplitude and/or conduction velocity)	3/8 Axon impairment	3/3 Demyelination	0/2	1/2 Demyelination	0/1
*Radiological findings*					
Brain MRI	5/10 Ear of the lynx, TCC, non-specific white matter lesions, cerebellar atrophy	3/5 Periventricular hyper-intensity, reversible hypersignals	3/3 Hyperintensities in corpus callosum, periventricular area, and corticospinal tract	2/2 Cerebellar atrophy, abnormal signals in the pons (ARSACS)	–
Spinal MRI	0/15	0/3	0/4	0/1	–

*AAO =* Age at onset, *TCC =* Thinning corpus callosum

**Table 2:** The detailed clinical information of 28 cases

Case	Gender	Age/ AAO	Family history	Initial symptoms	Pure spastic paraplegia					Other symptoms				
					Hypertonia	Reflex	Pyramidal sign	Deep sensibility	Foot deformity	Cerebral Sign	Extra-pyramidal	Dementia	Dysarthria	Peripheral neuropathy
					UL/LL	UL/LL	UL/LL							
1	M	18/12	–	GD	**−/+**	**−/+**	**−/+**	**–**	**–**	**–**	**–**	**–**	**–**	**–**
2	M	24/20	–	SL	**−/+**	**−/+**	**−/+**	**+**	**–**	**–**	**–**	**–**	**–**	**–**
3	F	14/4	+	GD	**−/+**	**−/+**	**−/+**	**–**	**+**	**–**	**–**	**–**	**–**	**–**
4	F	13/3	+	GD	**−/+**	**−/+**	**−/+**	**–**	**–**	**–**	**–**	**–**	**–**	**–**
5	M	43/13	+	GD	**−/+**	**−/+**	**−/+**	**+**	**–**	**–**	**–**	**–**	**–**	**+**
6	M	31/24	–	GD	**−/+**	**−/+**	**−/+**	**–**	**–**	**–**	**–**	**–**	**–**	**–**
7	F	47/27	+	GD	**−/+**	**−/+**	**−/+**	**–**	**–**	**–**	**–**	**–**	**–**	**+**
8	F	62/32	+	GD	**−/+**	**−/+**	**−/+**	**–**	**–**	**–**	**–**	**–**	**–**	**–**
9	F	44/4	–	WL	**−/+**	**−/+**	**−/+**	**–**	**–**	**–**	**–**	**–**	**–**	**–**
10	M	14/4	–	GD	**−/+**	**−/+**	**−/+**	**–**	**–**	**–**	**–**	**–**	**–**	**–**
11	M	21/17	+	GI/WL	**−/+**	**−/+**	**−/+**	**–**	**+**	**–**	**–**	**–**	**–**	**–**
12	F	22/20	–	GD	**−/+**	**−/+**	**−/+**	**–**	**–**	**+**	**–**	**–**	**–**	**+**
13	M	16/13	U	GI/D	**−/+**	**+/+**	**−/+**	**+**	**–**	**+**	**–**	**+**	**+**	**–**
14	F	39/37	–	WL	**−/+**	**+/+**	**−/+**	**+**	**–**	**–**	**–**	**–**	**+**	**–**
15	M	33/3	–	GI	**−/+**	**+/+**	**+/+**	**+**	**+**	**+**	**–**	**–**	**+**	**–**
16	F	13/13	+	GD	**−/+**	**−/+**	**−/+**	**–**	**+**	**+**	**–**	**+**	**+**	**+**
17	M	40/34	–	GD	**−/+**	**−/+**	**+/+**	**–**	**–**	**–**	**–**	**–**	**–**	**+**
18	M	20/13	–	GD	**−/+**	**+/+**	**+/+**	**–**	**–**	**–**	**–**	**–**	**–**	**–**
19	M	15/15	–	GD	**−/+**	**−/+**	**−/+**	**–**	**+**	**–**	**–**	**–**	**–**	**–**
20	M	23/23	–	WL	**−/+**	**−/+**	**−/+**	**–**	**+**	**–**	**–**	**–**	**–**	**+**
21	F	29/29	–	GD	**−/+**	**+/+**	**+/+**	**–**	**–**	**–**	**–**	**–**	**–**	**–**
22	M	32/30	+	GI/A	**−/+**	**+/+**	**+/+**	**–**	**–**	**–**	**–**	**+**	**+**	**–**
23	M	32/31	–	WL	**−/−**	**+/+**	**+/+**	**–**	**–**	**–**	**–**	**–**	**–**	**–**
24	M	24/24	–	GD	**−/+**	**−/+**	**−/+**	**–**	**–**	**–**	**–**	**–**	**–**	**–**
25	M	68/61	–	WL	**−/+**	**+/+**	**+/+**	**–**	**–**	**–**	**–**	**–**	**–**	**–**
26	F	30/13	+	GI/A	**−/+**	**+/+**	**+/+**	**–**	**+**	**+**	**–**	**–**	**+**	**–**
27	M	23/3	–	GI/A	**−/+**	**−/+**	**+/+**	**–**	**+**	**+**	**–**	**+**	**+**	**+**
28	M	14/12	–	GD	**−/+**	**−/+**	**−/+**	**–**	**+**	**–**	**–**	**–**	**–**	**–**

*M =* Male, *F =* Female, *U =* Unknown, *UL =* Upper limbs, *LL =* Lower limbs, *U =* Unknown, *AAO =* Age at onset, *GD =* Gait disturbance, *GI =* Gait instability, *WL =* Weakness of legs, *SL =* Stiffness of legs, *D =* Dementia, *A =* Alalia

### Hereditary spastic paraplegia

The subtypes in the group included SPG3A/*ATL1* (1/15)、SPG4/*SPAST* (4/15)、SPG5A/ *CYP7B1* (2/15)、SPG10/*KIF5A* (2/15)、SPG11/*SPG11* (2/15)、SPG15/*ZFYVE26* (1/15)、SPG31/*REEP1* (1/15)、SPG35/*FA2H* (1/15) and SPG46/*GBA2* (1/15). The mean age was 29.4 years (13–62 years), the average age at onset (AAO) was 15.5 years (3–37 years), and the duration ranged from 2 years to 40 years. Except for case 13 complain of gait instability with reduced intelligence, the rest of patients complained of gait disturbance, gait instability, stiffness, or weakness of the legs. There were eight cases of ADHSP and seven ARHSP. Among the ADHSP indexes, all of them had spasm of lower limbs, and six cases were pure forms and two were complicated. In ARHSP patients, all patients had spasm of lower limbs, 3/7 patients (case 13–15) had hyperreflexia of the upper limbs, and case 15 had positive Hoffmann’s sign. Two cases performed in pure forms and five cases in complicated. The additional neurological symptoms in the seven complicated patients (case 5, 7, 11–15) included peripheral neuropathy (3/7, case 5, 7 and 12), dysarthria (3/7, case 13–15), cerebellar signs (ataxia, nystagmus) (3/7, case 12, 13 and 15), dementia (1/7, case 13), and abnormal cerebral MRI (5/7, case 11–15).

Serum Hcy of case 6 and case 13 were 21.1 and 25.5 umol/L (normal value ≤15umol/L), and vitamin B12 were 133.5 and 132.4 pg/mL (normal value, 180–900 pg/mL), respectively. The rest of the patients were with normal serum Hcy, folic acid and vitamin B12. 8/9 cases showed prolonged central conduction in SEP, suggesting the involvement of the posterior cord. NCS was abnormal in 3/8 cases (case 5, 7 and 12), with decreased amplitude in case 5 and 12, and both declined amplitude and conduction velocity in case 7 (case 7 had a history of diabetes for 3 years). Case 1 and 13 had extensive spontaneous potentials (involved in at least three of four segments: medulla oblongata, cervical, thoracic and lumbar marrow) without abnormal NCS, suggesting possible involvement of the spinal anterior horn. Brain MRI was performed on 10 patients. “Ear of the lynx” and thinning corpus callosum were observed in the brain MRI of case 11/SPG11, 12/SPG11 and 13/SPG15. Case 14 showed hyperintensity in the white matter, and enlargement of the pituitary gland. Enhanced pituitary MRI showed no intensification. Brain MRI of case 15/SPG46 showed cerebellar atrophy. There was no significant change in brain MRI of the remaining patients.

### Hcy remethylation disorders

Three cases of MMA/*MMCHC* and two cases of MTHFR deficiency/ *MTHFR* were confirmed. The mean age was 22.2 years, and the average AAO was 19.6 years. The duration of three patients (case 16, 19 and 20) ranged from 2 to 5 months, and case 17 and 18 had a course of 6 and 7 years, respectively. The complains were gait disturbance (4/5) and weakness of legs (1/5). All patients had spasm of lower limbs, and 2/5 (case 17 and 18) had positive Hoffmann’s sign, and case 18 had hyperreflexia of the upper limbs. The additional neurological symptoms included peripheral neuropathy (3/5), dysarthria (1/5), cerebellar signs (1/5) and dementia (1/5). All cases presented with asymmetry of lower limbs spasm or weakness (not listed in the table). In addition, case 16 was readmitted 8 years later due to progressive mental decline and 1 month of psychiatric symptoms. Case 17 was previously admitted to the psychiatric department for bipolar disorder and accepted long-term antipsychotic medication. Serum Hcy was significantly increased in all patients ranging from 53.1–154 umol/L. Serum folate and vitamin B12 levels were normal. SEP displayed prolonged central conduction in 3/3 cases (case 16, 17 and 20), and conduction velocity of the 3 cases were decreased, suggesting the presence of demyelination. In case 16 and 20, the hypersignals were found in the terminal areas of white matter in brain MRI, suggesting delayed/impaired myelination. Periventricular hyperintensity was observed in case 16 and 17. In addition, reversible hypersignals were observed in the dorsal cerebellar hemisphere in case 16. No abnormalities were observed in case 18 and 19.

### Leukodystrophy

The five cases in the group were CTX/*CYP27A1* (1/5), X-ALD/*ABCD1* (2/5), HLD7/ *POLR3A* (1/5) and Krabbe’s disease (1/5). The average age was 37 years, and the mean AAO was 35 years. The course of the disease ranged from 5 months to 10 years. The complains were gait disturbance (2/5), lower limbs weakness (2/5), and gait instability with alalia (1/5). All patients had spasm of lower limbs, and 4/5 patients had hyperreflexia and Hoffmann’s sign of the upper limbs. Except for case 22 presented as complicated form, the rest had no additional neurological signs. β-galactocerebrosidase enzyme of case 25 was 2.6 nmol/17 h/mgPr, and the reference range was 19–68.2 nmol/17 h/mgPr. Folic acid, vitamin B12, and serum Hcy were normal in all patients. SEP showed abnormalities of central segment in 2/4 cases (case 23 and 25), but EMG and NCS were normal in the two patients. The remaining patients were not examined. Hyperintensities were found in the bilateral deep white matter, corpus callosum and internal capsules (case 22) and around the left anterior horn of the ventricle (case 23). There were hypersignals in the bilateral corticospinal tract area and the terminal areas of white matter in brain MRI of case 25.

### Hereditary ataxia

The onset age of case 26 (SCAR8/*SYNE1*) was 13 years old and case 27 (ARSACS/*SACS*) was 3 years old, and the age of admission was 30 and 23 years old respectively. Both patients complained of gait instability with alalia, and were characterized by complicated forms, with typical and prominent cerebellar signs. Case 26 was also accompanied by lingualis fibrillation and lower limbs weakness, as well as hyperreflexia and Hoffmann’s sign of the upper limbs. Case 27 walked with a spastic gait at the first visit, but the spasm of lower limbs was weakened in the follow-up, while Babinski’s and Hoffmann’s signs were always positive. No positive laboratory results were found in the HA group. EMG of case 26 showed spontaneous potentials (medulla oblongata, cervical, thoracic and lumbar marrow involved) without abnormal conduction velocity, and SEP suggested prolonged central conduction. Case 27 manifested demyelinating neuropathy of lower limbs, but SEP was not performed (Nerve biopsy displayed chronic demyelination changes, consistent with NCS). Both patients presented with cerebellar atrophy in the brain MRI. Brain MRI of case 27 showed T2-hypointense stripes at the central pons and diffuse T2-hyperintense areas at the lateral pons merging into thickened middle cerebellar peduncles in addition to superior vermis atrophy.

### Charcot-Marie-Tooth atrophy

The case 28 with CMTX4/*AIFM1* started at 12 years with complain of gait disturbance. Further analysis revealed lower limb spasticity, gastrocnemius atrophy, and foot deformity without abnormal audition. EMG of the patient showed only extensive spontaneous potentials (cervical, thoracic and lumbar marrow involved) with more severity in lower limbs and without abnormalities in NCS and SEP. No positive findings were found in laboratory tests.

It should be noted that no spinal abnormalities were found in all subjects.

### Molecular genetics analyses

**Fig. 2:**
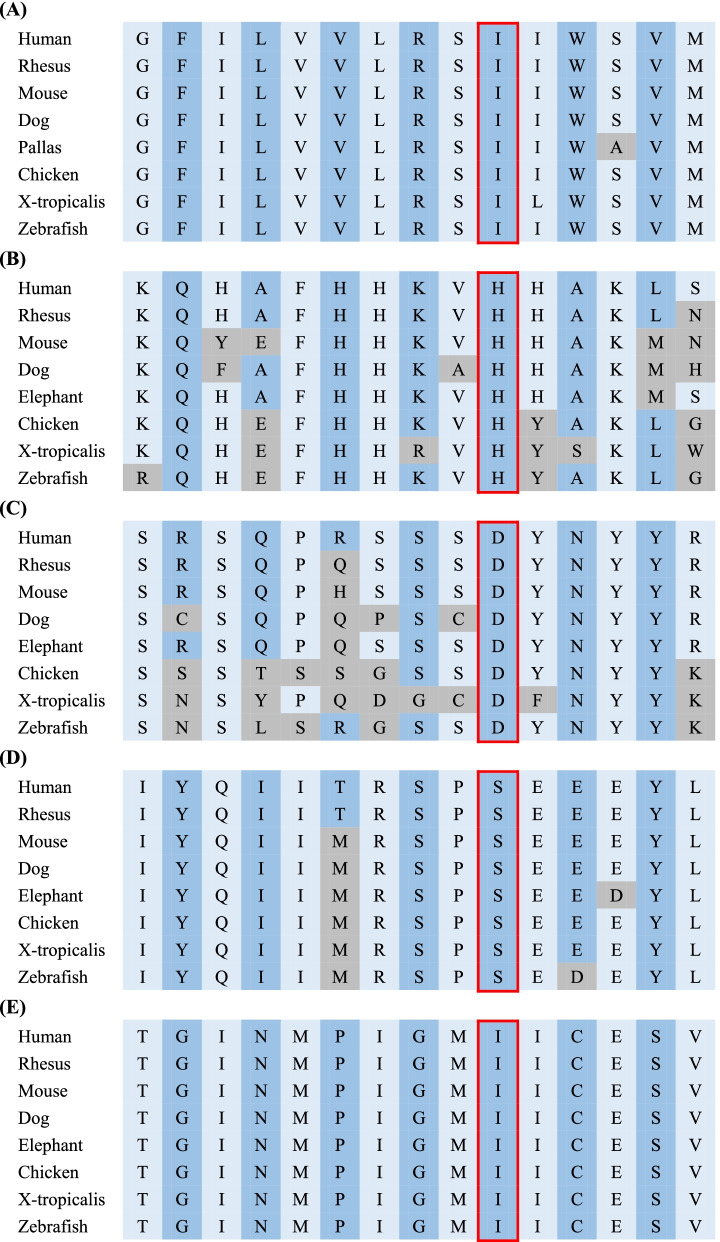
Conservation analysis of five missense mutations among species. A. REEP1 c.17 T > A, p.I6N B. FA2H c.1006C > G, p.H336D C. GBA2 c.2215G > T, p.D739Y D. MTHFR c.1808C > T, p.S603F E. POLR3A c.4044C > G, p.I1348M

The genetic data of 27 patients were described in detail in Table 3. Twelve new variants were identified, of which *SPAST*/c.891_892insG, *SPAST*/c.1140delC, *CYP7B1*/c.1512delG, *SPG11*/c.3022delT, *ZFYVE26*/c.6336C > G, *POLR3A*/c.2422C > T, and *SYNE1*/c.20485G > T were frameshift mutations or nonsense mutations, and *REEP1*/c.17 T > A, *FA2H*/c.1006C > G, *GBA2*/c.2215G > T, *MTHFR*/c.1808C > T, and *POLR3A*/c.4044C > G were missense mutations. According to the standards of ACMG, the frameshift or nonsense mutations were pathogenic or likely pathogenic, while the five missense mutations were uncertain for pathogenicity. Conservation analysis was performed on the five new missense mutations, and we found that they were all located in highly conservative sequences (Fig. 2). Combined with the clinical manifestations of the patients, we considered that they might be responsible lesions, and we would further conduct functional verification if possible. In addition, for the case 24/HLD7, whether the variant c.4044C > G was pathogenic should be further evaluated, and follow-up was necessary. Although the patients with these novel variants did not exhibit new phenotypes, mutational spectrum of these diseases was expanded.

**Table 3:** The genetic analysis of 27 cases

Case	Sex/AAO	Subtype	Gene	Chromosomal location	Transcript	Variant	Protein	Hom/het	Inheritance	ACMG	References for reported variants
1	M/12	SPG3A(P)	ATL1	chr1451081124	NM_015915	c.757G > A	p.V253I	het	AD	P	[12]
2	M/20	SPG4(P)	SPAST	chr2–32,339,776- 32,339,776	NM_014946	c.753dupA	p.V252SfsTer13	het	AD	LP	[13]
3	F/4	SPG4(P)	SPAST	chr2–32,379,455	NM_014946	c.1741C > T	p.R581X	het	AD	P	[14]
4	F/3	SPG4(P)	SPAST	chr2–32,379,455	NM_014946	c.1741C > T	p.R581X	het	AD	P	[14]
5	M/13	SPG4(C)	SPAST	chr2–32,340,789	NM_014946	c.891_892insG^a^	p.T298DfsTer3	het	AD	LP	–
6	M/24	SPG4(P)	SPAST	chr2:32352057–32,352,058	NM_014946	c.1140delC^a^	p.F381LfsTer15	het	AD	LP	–
7	F/27	SPG10(C)	KIF5A	chr12–57,961,297	NM_004984	c.610C > T	p.R204W	het	AD	LP	[15]
8	F/32	SPG31(P)	REEP1	chr2–86,564,617	NM_022912	c.17 T > A^a^	p.I6N	het	AD	U	–
9	F/4	SPG5A(P)	CYP7B1	chr8–65,517,390 chr8–65,536,958	NM_004820	c.1082G > A c.259 + 2 T > C	p.R361Q Splicing	het	AR	LP/P	[16, 17]
10	M/4	SPG5A(P)	CYP7B1	chr8–65,509,207-65,509,208 chr8–65,536,958	NM_004820	c.1512delG^a^ c.259 + 2 T > C	p.V504fs Splicing	het	AR	P/P	[17]
11	M/17	SPG11(C)	SPG11	chr15–44,907,576-44,907,577 chr15–44,955,591	NM_025137	c.3022delT^a^ c.255G > A	p.Y1008TfsTer29 p.W85X	het	AR	LP/P	[18]
12	F/20	SPG11(C)	SPG11	chr15–44,856,737-44,856,741 chr15–44,951,490-44,951,490	NM_025137	c.7151 + 4_7151 + 7delAGTA c.453dupA	Splicing p.L152IfsTer10	het	AR	LP/P	[19, 20]
13	M/13	SPG15(C)	ZFYVE26	chr14–68,228,953	NM_015346	c.6336C > G^a^	p.Y2112X	hom	AR	LP	–
14	F/37	SPG35(C)	FA2H	chr16–74,750,278 chr16–74,753,052	NM_024306	c.1006C > G^a^ c.620C > T	p.H336D p.T207M	het	AR	U/LP	[8]
15	M/3	SPG46(C)	GBA2	chr9–35,737,315 chr9–35,738,132	NM_020944	c.2635C > T c.2215G > T^a^	p.R879W p.D739Y	het	AR	LP/U	[6]
16	F/13	MMACHC	MMACHC	chr1–45,974,520 chr1–45,974,693-45,974,696	NM_015506	c.482G > A c.656_658del	p.R161Q p.219_220del	het	AR	P/P	[21]
17	M/34	MMACHC	MMACHC	chr1–45,974,520 chr1–45,974,647	NM_015506	c.482G > A c.609G > A	p.R161Q p.W203 X	het	AR	P/LP	[21]
18	M/13	MMACHC	MMACHC	chr1–45,974,482-45,974,484 chr1:45974520	NM_015506	c.445_446delTG c.482G > A	p.C149HfsTer32 p.R161Q	het	AR	P/P	[21, 22]
19	M/15	MTHFR	MTHFR	chr1:11855183 chr1:11850900	NM_005957	c.1003C > T c.1808C > T^a^	p.R335C p.S603F	het	AR	LP/U	[23]
20	M/23	MTHFR	MTHFR	chr1–11,856,345 chr1–11,863,038	NM_005957	c.698C > G c.136C > T	p.A233G p.R46W	hom	AR	LP	[24, 25]
21	F/29	CTX	CYP27A1	chr2–219,674,454 chr2–219,679,132	NM_000784	c.410G > A c.1214G > A	p.R137Q p.R405Q	het	AR	LP/LP	[26, 27]
22	M/30	X-ALD	ABCD1	chrX-153,002,631-153,002,633	NM_000033	c.1415_1416del	p.Q472RfsTer82	hemi	XLR	P	[28]
23	M/31	X-ALD	ABCD1	chrX:152994860–152,994,860	NM_000033	c.1074dupA	p.E359RfsTer42	hemi	XLR	P	[29]
24	M/24	HLD7	POLR3A	chr10–79,760,790 chr10–79,737,365	NM_007055	c.2422C > T^a^ c.4044C > G^a^	p.R808X p.I1348M	het	AR	LP/U	–
25	M/61	Krabbe’s D	–	–	–	–	–	–	–	–	–
26	F/13	SCAR8	SYNE1	chr6–152,554,930	NM_033071	c.20485G > T^a^	p.E6829X	hom	AR	LP	–
27	M/3	ARSACS	SACS	chr13–23,905,337-23,905,342	NM_014363	c.12673_12677del	p.Y4225DfsTer5	hom	AR	LP	[30]
28	M/12	CMTX4	AIFM1	chX-129,271,098	NM_004208	c.1030C > T	p.L344F	hemi	XLR	P	[31]

^a^The new variants . *P = *Pathogenic, *LP =* Likely pathogenic, *U =* Uncertain

## Discussion

### Clinical features

The average duration from onset to diagnosis in the HSP group was remarkably longer than that in the Hcy remethylation disorders group and LD group, suggesting a slow progression. In fact, CTX, X-ALD, Krabbe’s disease and Hcy remethylation disorders were hereditary metabolic diseases, and metabolic processes were susceptible to many factors, even with acute or subacute outbreak occasionally due to accumulation of metabolites [32]. Upper motor neuron signs involved the upper limbs in 4/5 cases of the LD group and 2/5 of the Hcy remethylation disorders group and 2/2 of the HA group, which were different from the cases of the HSP group (3/15). In general, proteins encoded by HSP genes have diverse functions including axon growth and transport, endoplasmic reticulum morphology, mitochondrial function, myelination, lipid and sterol metabolism, endosome/lysosome dynamics, and autophagy. Long spinal axons are likely to be susceptible to perturbations of membrane trafficking and axonal transport, leading to abnormal axonal growth and maintenance, and eventually to degeneration. As a result, neurodegeneration starts at the distal ends of axons; thereby the lower extremities are affected first [33–35]. Postmortem studies consistently identified degeneration of corticospinal tract and fasciculus gracilis fibers, maximal in the thoracic and cervical spinal cord respectively, which was more severe at distal ends [9, 36]. Demyelination/hypomyelination in the nervous system played a vital role in pathophysiology process of Hcy remethylation disorders and LD [32, 37–40]. Different from significant length-dependent features on the metabolism of axon, the myelination was performed by oligodendrocytes (central nervous system) and Schwann cells (peripheral nervous system), and function of myelin was maintained mainly by lipid metabolism. Thereby, upper motor neuron signs were more likely to occur in the four extremities. The pathology of adrenomyeloneuropathy (AMN) was characterized predominantly by a distal axonopathy involving the long tracts of the spinal cord, which was fundamentally different from that of cerebral ALD, resulting in a progressive spastic paraplegia [41]. Case 26/SCAR8 and case 27/ARSACS could present with spastic paraplegia initially, because of HA sharing many pathophysiological mechanisms with HSP [42]. However, the data in this study suggested that the upper limbs of HA patients were more likely affected than those of HSP patients. Furthermore, instead of occurring after a long period of spastic gait in HSP patients, cerebellar signs in HA patients appeared earlier and more remarkable. Interestingly, patients with Hcy remethylation disorders were all characterized by asymmetry spasm or weakness of lower limbs, as well as in case 25/Krabbe’s diseases, which was not spelt out in previous researches. Many causes, like infection, could speed up the disordered metabolic processes, resulting in an acute episode. But with treatment or the removal of the causes, the disorder was corrected and the damage of central nervous system was gradually restored. These reversible processes may lead to bilaterally asymmetric and unstable accumulation of metabolic substrates. In addition, case 26/SCAR8 was accompanied by obvious lower motor neuron signs such as lingualis fibrillation and lower limbs weakness, with extensive spontaneous potentials (sternocleidomastoid, lingualis, thoracic paravertebral muscles, upper and lower limb muscles) in EMG. Studies have shown that mutations in the C-terminal region of SYNE1 could be associated with lower motor neuron signs, due to the abnormal expression of the short isoforms of nesprin-1 causing motor neuron dysfunction [43]. The variant p.E6829X was exactly located at C-terminal. Verrips et al. [44] described that spinal cord syndrome existed for many years before the classic symptomatology became manifest in CTX, and tendinoxanthoma occurred in 71% of patients. Therefore, CTX should be considered as a differential diagnosis of HSP, especially in the absence of symptoms of tendinoexanthoma (case 21). Inspite of the fact that there was at least one change of the clinical features (neurological, dental, ophthalmic, and endocrine changes) in every HLD7 patient, case 24 only had spinal cord manifestations, so the variants of the patient need to be further verified for its pathogenicity, and follow-up was also very necessary.

### Laboratorial features

Laboratory tests can often be diagnostic in the inherited metabolic diseases. An elevated serum Hcy is the common biochemical marker in Hcy remethylation disorders. Serum Hcy of all MMA/MHTFR deficiency patients was higher than 50 umol/L in the study. The level of β-galactocerebrosidase enzyme decreased markedly in case 25, indicating the Krabbe’s disease. Adrenal function and very-long-chain fatty acids (VLCFA) tests are necessary when HSP is considered, especially in the X-linked mode of inheritance.

### Electrophysiological features

In the HSP group, 3 out of 8 patients (case 5, 7, and 12) had declined amplitude, and case 7 had decreased conduction velocity, suggesting that the peripheral neuropathy was mainly axonal. In the MMA/MTHFR deficiency group, 3/3 patients (case 16, 17, and 20) showed demyelinating neuropathy, resulting from abnormalities in the synthesis of the myelin sheath [32]. In the LD group, although there was no evidence of neuropathy on neuro-electrophysiology examination, several studies reported that peripheral neuropathy in CTX could be divided into three types: axonal, myelinated and mixed forms; while that in X-ALD was mixed, and myelinated in Krabbe’s disease [40, 45–47]. EMG of the case 26/SCAR8 showed extensive spontaneous potentials of four spinal segments without abnormal nerve conduction, suggesting damage to the anterior horn of spinal cord, as explained above. Axonal neuropathy was reported in the literatures, but, peripheral neuropathy is a rather infrequent feature in SYNE1 disease [48–50]. In this study, case 27 was accompanied by demyelinating neuropathy. ARSACS was traditionally described as a trilogy, ataxia, pyramidal involvement, and axonal neuropathy. However, a series of researches reported both axon and myelin sheath damage. Interestingly, CMTX4 was charactered by an early-onset axonal sensorimotor neuropathy and hearing loss with additional features including spasticity, cognitive impairment, and cerebellar signs, as well as cerebellar atrophy in brain imaging [51]. Also, some cases of motor neuron involvement have been reported, emphasizing the role of AIFM1 in the development and function of motor neurons [52]. Case 28 presented as typical spastic paraplegia and gastrocnemius atrophy with extensive spontaneous potentials, which was different from the reported phenotypes.

### Radiological features

In the HSP group, “ear of the lynx” and thinning corpus callosum are common and characteristic findings for SPG11 (case 11 and 12) and SPG15 (case 13), especially for SPG11 [5, 8], and non-specific white matter lesions (case 14/SPG35) and cerebellar atrophy (case 15/SPG46) have been described. But no pituitary enlargement (case 14/SPG35) has been reported, and the relationship with the disease is unknown. The common manifestations in brain MRI of adult-onset Krabbe’s disease were hyperintensities in the area of the pyramidal tract, periventricular, parieto-occipital region, and posterior corpus callosum, among which only involvement of the area of the pyramidal tract accounted for 75%. The cerebellum, basal ganglia, and thalamus were almost not involved [53, 54]. Case 25 had the typical characteristics of pyramidal tract involvement. It’s worth noting that the presence of marked hypersignals in corticospinal tract pathways is a common finding in different pure or complicated HSP [7], but the difference is that the abnormal signals in HSP rarely extend to the anterior gyrus of the cortex. Brain MRI in case 27/ARSACS showed characteristic hypersignals in the pons, which occurred in > 90% of patients and were highly helpful diagnostic imaging markers [55]. In addition, reversible signals in brain MRI could appear in MMA/MHTFR deficiency patients, such as case 16.

However, spinal cord thinning did not exist in any of our patients. It should be noted that volume analysis (measuring spinal cord cross-sectional area at stages C2 and T9) was not performed according to the criteria described by Hedera et al. [56], but by visual evaluation from experienced neuroimaging physicians. In addition, part of the cranial MRI changes and spinal cord atrophy may be late events during disease.

## Conclusion

HSP had widespread clinical and genetic heterogeneity, and LD, HA, CMT, and Hcy remethylation disorders accounted for a significant proportion of the proposed HSP. These hereditary neurological diseases had different characteristics in clinical, laboratorial, electrophysiological, and radiological aspects, which could help differential diagnosis. When the aforementioned methods fail to yield a clear diagnosis, extended genetic analysis spectrum could be used as a reliable diagnostic tool.

## Supplementary Information

**Additional file 1.**

## Data Availability

All data generated or analysed during this study are included in this published article [and its supplementary information files].

## Abbreviations

AAO: Age at onset
AD: Autosomal dominant
ALS: Amyotrophic lateral sclerosis
AMN: Adrenomyeloneuropathy
AR: Autosomal recessive
ARSACS: Autosomal recessive spastic ataxia of Charlevoix-Saguenay
CMT: Charcot-Marie-Tooth atrophy
CMTX4: X-linked Charcot-Marie-Tooth disease type 4
CTX: Cerebrotendinous xanthomatosis
HA: Hereditary ataxia
Hcy: Homocysteine
HLD-7: Hypomyelination leukodystrophy-7
HSP: Hereditary spastic paraplegia
LD: Leukodystrophy
MD: Movement disorder
MMA: Methylmalonic acidemia
MTHFR: Methylenetetrahydrofolate reductase
NCS: Nerve conduction studies
NGS: Next-generation sequencing
SCA: Spinocerebellar ataxia
SCAR8: Autosomal recessive cerebellar ataxia type 8
SEP: Sensory evoked potentials
TCC: Thinning corpus callosum
X-ALD: X-linked adrenoleukodystrophy
