# Supplementary material for: Genetic origin of patients having spastic paraplegia with or without other neurologic manifestations
Source: BMC Neurol. 2022 May 16;22:180. doi: 10.1186/s12883-022-02708-z (PMC9109329; doi:10.1186/s12883-022-02708-z)
Supplement: Supplementary file 1 — Additional file 1. [file 12883_2022_2708_MOESM1_ESM.docx]

Table 1 Genes of HSP-associated panel

| PLP1 | SPG7 | KIF5A | DDHD1 | C19orf12 | CYP2U1 | AMPD2 | MARS | BICD2 |
| --- | --- | --- | --- | --- | --- | --- | --- | --- |
| ATL1 | SPAST | CYP7B1 | NIPA1 | WASHC5 | RTN2 | HSPD1 | REEP1 | ZFYVE27 |
| SLC33A1 | USP8 | ERLIN1 | REEP2 | ADAR | IFIH1 | RNASEH2B | L1CAM | SPG11 |
| ZFYVE26 | BSCL2 | ERLIN2 | SPG20 | SPG21 | SLC16A2 | B4GALNT1 | KIF1A | FA2H |
| PNPLA6 | GJC2 | NT5C2 | GBA2 | AP4B1 | AP5Z1 | TECPR2 | AP4M1 | AP4E1 |
| AP4S1 | VPS37A | DDHD2 | C12orf65 | TFG | KIF1C | WDR48 | ARL6IP1 | ENTPD1 |
| NT5C2 | ARSI | PGAP1 | FLRT1 | RAB3GAP2 | ZFR | IBA57 | EXOSC3 | FAM134B |
| LYST | MAG | CPT1C | VCP | CCT5 | CAPN1 | ALDH18A1 |  |  |

Table 2 Genes of movement disorder panel

| AAAS | AARS2 | ABCB7 | ABCD1 | ABHD12 | ACACA | ACO2 | ADAR |
| --- | --- | --- | --- | --- | --- | --- | --- |
| ADCY5 | ADGRG1 | ADH1C | AFG3L2 | AHI1 | AIFM1 | ALDH18A1 | ALDH3A2 |
| ALDH5A1 | ALG1 | ALG11 | ALG12 | ALG13 | ALG2 | ALG3 | ALG6 |
| ALG8 | ALG9 | ALS2 | AMACR | AMPD2 | ANO10 | ANO3 | AP4B1 |
| AP4E1 | AP4M1 | AP4S1 | AP5Z1 | APOB | APOE | APP | APTX |
| ARG1 | ARL13B | ARL6IP1 | ARSA | ARSI | ARX | ASL | ASS1 |
| ATAD3A | ATCAY | ATG5 | ATL1 | ATM | ATN1 | ATP13A2 | ATP1A2 |
| ATP1A3 | ATP2B3 | ATP2B4 | ATP6AP2 | ATP7A | ATP7B | ATP8A2 | ATRX |
| ATXN1 | ATXN10 | ATXN2 | ATXN3 | ATXN7 | AUH | B4GALNT1 | BCKDHA |
| BCKDHB | BCKDK | BCS1L | BEAN1 | BICD2 | BSCL2 | BST1 | BTD |
| C12orf65 | C19orf12 | C9orf72 | CA8 | CACNA1A | CACNB4 | CAMTA1 | CAPN1 |
| CASK | CAV1 | CBS | CC2D2A | CCDC62 | CCDC88C | CCT5 | CEP290 |
| CEP41 | CHCHD10 | CHCHD2 | CHMP1A | CHMP2B | CIZ1 | CLCN2 | CLN5 |
| CLN6 | CLP1 | COASY | COL18A1 | COQ2 | COQ8A | COX10 | COX15 |
| COX20 | CP | CPLANE1 | CPS1 | CPT1C | CSF1R | CSPP1 | CSTB |
| CTC1 | CTDP1 | CTSA | CTSF | CUL4B | CWF19L1 | CYP27A1 | CYP2U1 |
| CYP7B1 | DAGLA | DARS | DARS2 | DBT | DCAF17 | DCTN1 | DDC |
| DDHD1 | DDHD2 | DDOST | DDRGK1 | DKC1 | DLD | DNAJC13 | DNAJC19 |
| DNAJC6 | DNM2 | DNMT1 | DOLK | DPAGT1 | DPM1 | DPM2 | DPM3 |
| DRD2 | DRD3 | DRD5 | DSTYK | DYNC1H1 | ECM1 | EEF2 | EIF2B1 |
| EIF2B2 | EIF2B3 | EIF2B4 | EIF2B5 | EIF4G1 | ELOVL4 | ELOVL5 | ELOVL7 |
| ENTPD1 | EPM2A | ERCC4 | ERCC6 | ERCC8 | ERLIN1 | ERLIN2 | ETFDH |
| ETHE1 | EXOSC3 | EXOSC8 | FA2H | FARS2 | FAT2 | FBXO47 | FBXO7 |
| FBXW7 | FGF14 | FLRT1 | FLVCR1 | FMR1 | FOLR1 | FOXRED1 | FTL |
| FUS | FUZ | FXN | GAD1 | GALC | GAN | GBA | GBA2 |
| GBE1 | GCDH | GCH1 | GCLC | GFAP | GIGYF2 | GJA1 | GJC2 |
| GJD2 | GLB1 | GLRX5 | GLUD2 | GM2A | GNAL | GOSR2 | GPNMB |
| GPT2 | GRID2 | GRM1 | GRN | HAPLN4 | HEPACAM | HEPH | HEXA |
| HEXB | HIBCH | HLCS | HMOX1 | HS1BP3 | HSD17B4 | HSPD1 | HTRA1 |
| HTRA2 | IBA57 | IDS | IDUA | IFIH1 | IFRD1 | IL1B | INPP5E |
| INPP5F | ITPR1 | KCNA1 | KCNA2 | KCNC3 | KCND3 | KCNJ10 | KCNS2 |
| KCTD7 | KIF1A | KIF1C | KIF5A | KIF7 | KLC2 | KLC4 | L1CAM |
| L2HGDH | LINGO1 | LMNB1 | LRPPRC | LRRK2 | LYST | MAG | MAN2B1 |
| MAPT | MARS | MARS2 | MC1R | MCCC1 | MECP2 | MFSD8 | MIR4697 |
| MKS1 | MLC1 | MMACHC | MMADHC | MPDU1 | MPI | MPV17 | MRE11 |
| MSTO1 | MTFMT | MTHFR | MTPAP | MTR | MTRR | MTTP | MUT |
| MVK | NAGS | NARS2 | NDUFA10 | NDUFAF2 | NDUFAF6 | NDUFS3 | NDUFS4 |
| NDUFS7 | NDUFS8 | NEU1 | NFU1 | NGLY1 | NHLRC1 | NIPA1 | NKX2-1 |
| NOP56 | NOS3 | NOTCH3 | NPC1 | NPC2 | NPHP1 | NR4A2 | NT5C2 |
| NUBPL | NUCKS1 | OFD1 | OPA1 | OPA3 | OPHN1 | OTC | PACRG |
| PANK2 | PARK7 | PAX6 | PC | PCCA | PCCB | PCLO | PDE6D |
| PDGFB | PDGFRB | PDHX | PDX1 | PDYN | PEX10 | PEX16 | PEX2 |
| PEX6 | PEX7 | PGAP1 | PGM1 | PHYH | PIK3R5 | PINK1 | PLA2G6 |
| PLD3 | PLEKHG4 | PLP1 | PMM2 | PMPCA | PNP | PNPLA6 | PODXL |
| POLG | POLR1C | POLR3A | POLR3B | PPP2R2B | PRF1 | PRICKLE1 | PRKCG |
| PRKN | PRKRA | PRNP | PRPS1 | PRRT2 | PRX | PSAP | PSEN1 |
| PSEN2 | PTRH2 | PTRHD1 | PTS | QDPR | RAB18 | RAB29 | RAB39B |
| RAB3GAP1 | RAB3GAP2 | RARS2 | REEP1 | REEP2 | RELN | RETREG1 | RFT1 |
| RIC3 | RNASEH2B | RNF168 | RNF170 | RNF216 | ROGDI | RPGRIP1L | RRM2B |
| RTN2 | RTN4IP1 | RUBCN | SACS | SARS2 | SCARB2 | SCN1A | SCN8A |
| SCTR | SDHA | SEPSECS | SERAC1 | SETX | SGCE | SIL1 | SIPA1L2 |
| SLC16A2 | SLC17A5 | SLC19A3 | SLC1A2 | SLC1A3 | SLC20A2 | SLC25A1 | SLC25A13 |
| SLC25A15 | SLC25A46 | SLC2A1 | SLC30A10 | SLC33A1 | SLC52A2 | SLC6A19 | SLC6A3 |
| SLC9A6 | SNCA | SNCAIP | SNCB | SNORD118 | SNX14 | SORT1 | SPART |
| SPAST | SPG11 | SPG21 | SPG7 | SPR | SPTBN2 | SQSTM1 | SRD5A3 |
| ST8SIA2 | STK39 | STT3A | STT3B | STUB1 | STX1B | SUOX | SURF1 |
| SYNE1 | SYNJ1 | SYT14 | TAF1 | TARDBP | TBC1D20 | TBP | TCTN1 |
| TCTN2 | TCTN3 | TDP1 | TECPR2 | TENM4 | TFG | TGM6 | TH |
| THAP1 | TIMM8A | TMEM138 | TMEM216 | TMEM230 | TMEM231 | TMEM237 | TMEM240 |
| TMEM67 | TOR1A | TOR1B | TPP1 | TRAPPC11 | TRMT5 | TRNT1 | TRPC3 |
| TRPM7 | TSEN2 | TSEN34 | TSEN54 | TTBK2 | TTC19 | TTPA | TUBB2A |
| TUBB4A | TWNK | UBE3A | UCHL1 | USP46 | USP8 | UVSSA | VAMP1 |
| VARS2 | VCP | VLDLR | VPS13A | VPS13C | VPS35 | VPS37A | VPS53 |
| VRK1 | VWA3B | WASF3 | WASHC5 | WDR45 | WDR48 | WDR73 | WDR81 |
| WFS1 | WWOX | XK | XRCC4 | ZFR | ZFYVE26 | ZFYVE27 | ZNF423 |
| ZNF592 |  |  |  |  |  |  |  |
